# Compartmentalized spatial profiling of the tumor microenvironment in head and neck squamous cell carcinoma identifies immune checkpoint molecules and tumor necrosis factor receptor superfamily members as biomarkers of response to immunotherapy

**DOI:** 10.3389/fimmu.2023.1135489

**Published:** 2023-04-03

**Authors:** Habib Sadeghirad, Ning Liu, James Monkman, Ning Ma, Bassem Ben Cheikh, Niyati Jhaveri, Chin Wee Tan, Majid Ebrahimi Warkiani, Mark N. Adams, Quan Nguyen, Rahul Ladwa, Oliver Braubach, Ken O’Byrne, Melissa Davis, Brett G. M. Hughes, Arutha Kulasinghe

**Affiliations:** ^1^ Frazer Institute, Faculty of Medicine, The University of Queensland, Brisbane, QLD, Australia; ^2^ Department of Bioinformatics Devision, The Walter and Eliza Hall Institute, Melbourne, VIC, Australia; ^3^ Department of Medical Biology, Faculty of Medicine, Dentistry and Health Sciences, University of Melbourne, Melbourne, VIC, Australia; ^4^ Akoya Biosciences, Menlo Park, California, CA, United States; ^5^ School of Biomedical Engineering, University of Technology, Sydney, NSW, Australia; ^6^ School of Biomedical Science, Faculty of Health, Queensland University of Technology, Brisbane, QLD, Australia; ^7^ Centre for Genomics and Personalised Health, Queensland University of Technology, Brisbane, QLD, Australia; ^8^ Institute of Molecular Biosciences, The University of Queensland, Brisbane, QLD, Australia; ^9^ The Princess Alexandra Hospital, Brisbane, QLD, Australia; ^10^ Department of Clinical Pathology, Faculty of Medicine, Dentistry and Health Sciences, University of Melbourne, Melbourne, VIC, Australia; ^11^ South Australian immunoGENomics Cancer Institute, The University of Adelaide, Adelaide, SA, Australia; ^12^ The Royal Brisbane and Women’s Hospital, Brisbane, QLD, Australia; ^13^ Faculty of Medicine, The University of Queensland, Brisbane, QLD, Australia

**Keywords:** spatial proteomics, head and neck cancer, tumor microenvironment, immunotherapy, head and neck squamous cell carcinoma (HNSCC)

## Abstract

Mucosal head and neck squamous cell carcinoma (HNSCC) are the seventh most common cancer, with approximately 50% of patients living beyond 5 years. Immune checkpoint inhibitors (ICIs) have shown promising results in patients with recurrent or metastatic (R/M) disease, however, only a subset of patients benefit from immunotherapy. Studies have implicated the tumor microenvironment (TME) of HNSCC as a major factor in therapy response, highlighting the need to better understand the TME, particularly by spatially resolved means to determine cellular and molecular components. Here, we employed targeted spatial profiling of proteins on a cohort of pre-treatment tissues from patients with R/M disease to identify novel biomarkers of response within the tumor and stromal margins. By grouping patient outcome categories into response or non-response, based on Response Evaluation Criteria in Solid Tumors (RECIST) we show that immune checkpoint molecules, including PD-L1, B7-H3, and VISTA, were differentially expressed. Patient responders possessed significantly higher tumor expression of PD-L1 and B7-H3, but lower expression of VISTA. Analysis of response subgroups indicated that tumor necrosis factor receptor (TNFR) superfamily members including OX40L, CD27, 4-1BB, CD40, and CD95/Fas, were associated with immunotherapy outcome. CD40 expression was higher in patient-responders than non responders, while CD95/Fas expression was lower in patients with partial response (PR) relative to those with stable disease (SD) and progressive disease (PD). Furthermore, we found that high 4-1BB expression in the tumor compartment, but not in the stroma, was associated with better overall survival (OS) (HR= 0.28, p-adjusted= 0.040). Moreover, high CD40 expression in tumor regions (HR= 0.27, p-adjusted= 0.035), and high CD27 expression in the stroma (HR= 0.2, p-adjusted=0.032) were associated with better survival outcomes. Taken together, this study supports the role of immune checkpoint molecules and implicates the TNFR superfamily as key players in immunotherapy response in our cohort of HNSCC. Validation of these findings in a prospective study is required to determine the robustness of these tissue signatures.

## Introduction

Mucosal head and neck squamous cell carcinoma (HNSCC) is one of the cancers that severely impact patients’ quality of life and causes complications such as pain, psychosocial distress, and dysfunction (1, 2). Tobacco and alcohol consumption are common risk factors for the development of SCC in the oral cavity, oropharynx, hypopharynx, and larynx (1, 3). Human Papilloma Virus (HPV) is also implicated in the pathogenesis of oropharyngeal SCC. Late diagnosis is common, with most HNSCC patients presenting with advanced disease (3). In the absence of distant metastasis, patients with advanced HNSCC are often treated with multimodality therapy such as surgery and radiotherapy and/or chemotherapy (3, 4). With recurrent disease in a previously irradiated site not amenable to salvage surgery, immunotherapy has shown modest benefits for patients with recurrent or metastatic (R/M) disease. Immune checkpoint inhibitors (ICIs) Pembrolizumab and Nivolumab are the FDA-approved treatments for HNSCC, particularly in patients with cisplatin-refractory R/M disease in the second line setting (3–7). More recently, Keynote 048 has shown a survival advantage of Pembrolizumab alone or with chemotherapy in the first-line setting depending on PD-L1 status. Nonetheless, it should be noted that only a minority of patients benefit from ICIs, making treatment of R/M HNSCC challenging (4).

**Figure 1:**
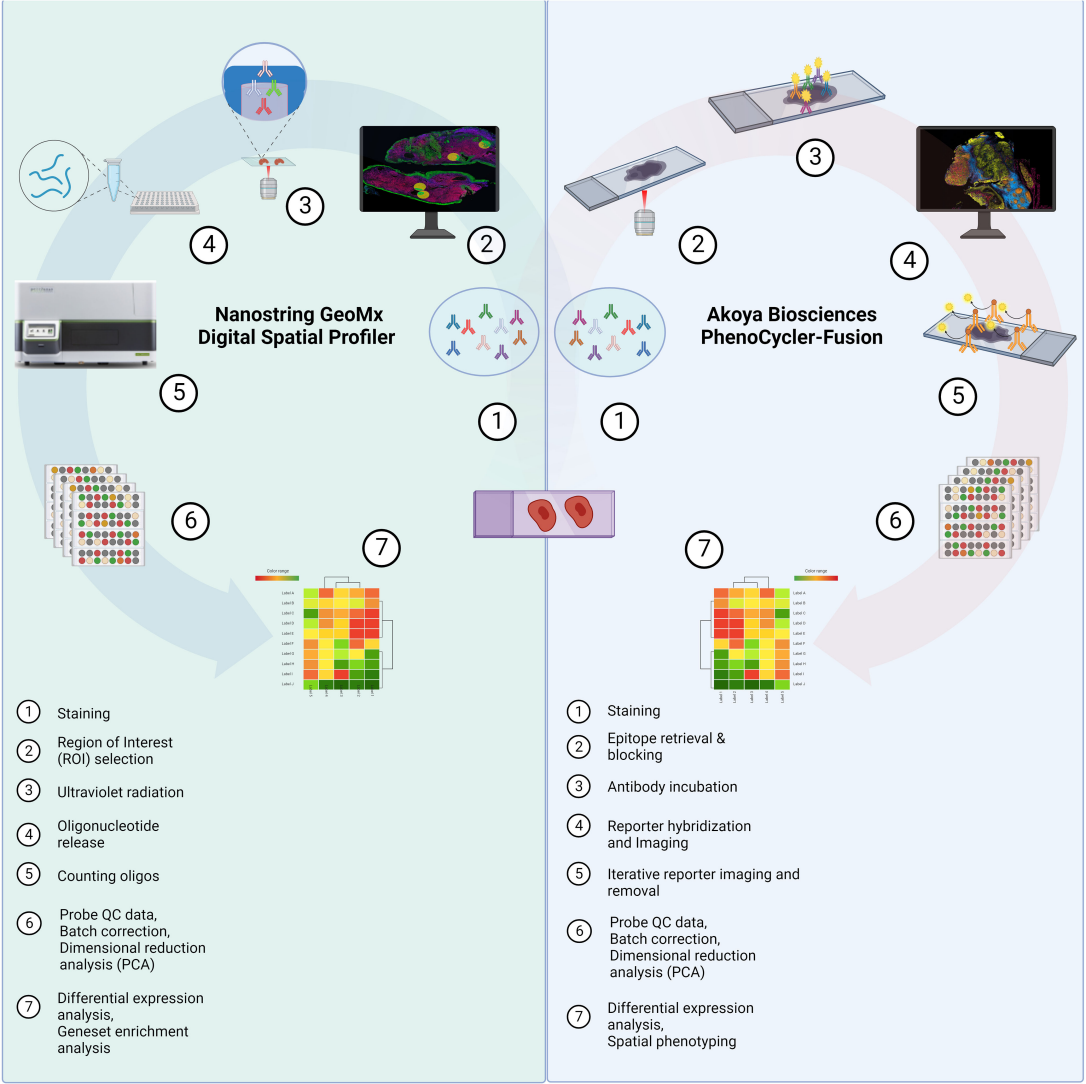
Study Schema. Formalin-fixed paraffin-embedded (FFPE) tissue samples were collected from HNSCC patients prior to immunotherapy from the Royal Brisbane and Women’s Hospital (RBWH). Targeted spatial proteomics across the cohort was performed with the Nanostring GeoMx Digital Spatial Profiler (DSP) and a sub-cohort analysis using the Akoya PhenoCycler-Fusion platform. Data analysis consisted of probe quality control (QC), principal component analysis (PCA), differential expression, and Cox proportional hazards (COXPH) and Kaplan-Meier models for survival analysis. Created by BioRender.com.

Studies have shown that the composition of the HNSCC tumor microenvironment (TME) and the interactions of immune cell types within the TME may be important determinants of treatment outcomes (2, 8). As a result, investigation of the HNSCC TME could lead to the identification of mechanisms underlying therapy response/resistance, paving the way for more personalized medicine (8). Expression profiling of immune biomarkers and the spatial phenotyping of cell types within the stroma and tumor compartments of HNSCCs can be accomplished using novel spatial profiling technologies (9), with the location and distance of immune cells, specifically cytotoxic T cells, to tumor cells found to be key factors for predicting treatment outcomes (10). In this study, we used the Nanostring Technologies GeoMx Digital Spatial Profiler (DSP) and the Akoya PhenoCycler-Fusion®, to explore the TME of HNSCC patients who received immunotherapy (**Figure 1**). Our goal was to identify protein biomarkers that are informative of immunotherapy outcomes.

## Material and methods

### Patient cohort

This retrospective study has Human Research Ethics (HREC) approval from the Royal Brisbane and Women’s Hospital (RBWH) (LNR/2020/QRBW/66744) and The University of Queensland ratification. We identified n=41 HNSCC patients eligible for our study from the RWBH. Of the 41 patients, 20 had no available tissues, with 21 tissue blocks available for analysis. We collected formalin-fixed paraffin-embedded (FFPE) tissue samples from these 21 HNSCC patients, with samples collected prior to immunotherapy. Pathology Queensland prepared serial sections and hematoxylin and eosin (H&E) staining, while pathology reviews provided demarcation of tumour/stromal regions so that non-neoplastic epithelium could be avoided. Of the 21 tissues collected, n=17 were deemed of appropriate quality and tissue integrity for subsequent spatial analysis. Patients were treated with Pembrolizumab or Nivolumab and categorized based on response to therapy according to RECIST 1.1., including complete response (CR), partial response (PR), stable disease (SD), and progressive disease (PD). The clinicopathological findings are shown in **Table 1**.

**Table 1:** HNSCC cohort characteristics.

Patients’ characteristics	
Characteristics	All patients (N=17)
Age, median (range)	68 (49-81)
Gender	
Male	14 (80%)
Female	3 (20%)
Smoking Status	
Current/former smokers	15 (90%)
Non-smokers	1 (5%)
Unknown	1 (5%)
ECOG performance status	
0	4 (25%)
1	13 (75%)
Status	
Alive	12 (70%)
Deceased	5 (30%)
Tumour location	
Base of tongue	4 (25%)
Unspecified parts of the tongue	2 (15%)
Retromolar area	1 (5%)
Tonsillar fossa	1 (5%)
Hard palate	1 (5%)
Tonsil	4 (25%)
Overlapping lesion of tongue	1 (5%)
Hypopharynx	1 (5%)
Unspecified parts of the mouth	1 (5%)
Postericoid region	1 (5%)
Immunotherapy	
Pembrolizumab monotherapy	3 (20%)
Pembrolizumab + chemotherapy	1 (5%)
Pembrolizumab + IDOI inhibitor	1 (5%)
Nivolumab monotherapy	12 (70%)
Best response	
Complete response (CR)	1 (5%)
Partial response (PR)	5 (30%)
Stable disease (SD)	3 (20%)
Progressive disease (PD)	8 (45%)
Oropharyngeal p16 status	
Positive	7 (40%)
Negative	3 (20%)
Unknown	7 (40%)

### Nanostring GeoMx digital spatial profiler (DSP)

**Figure 2:**
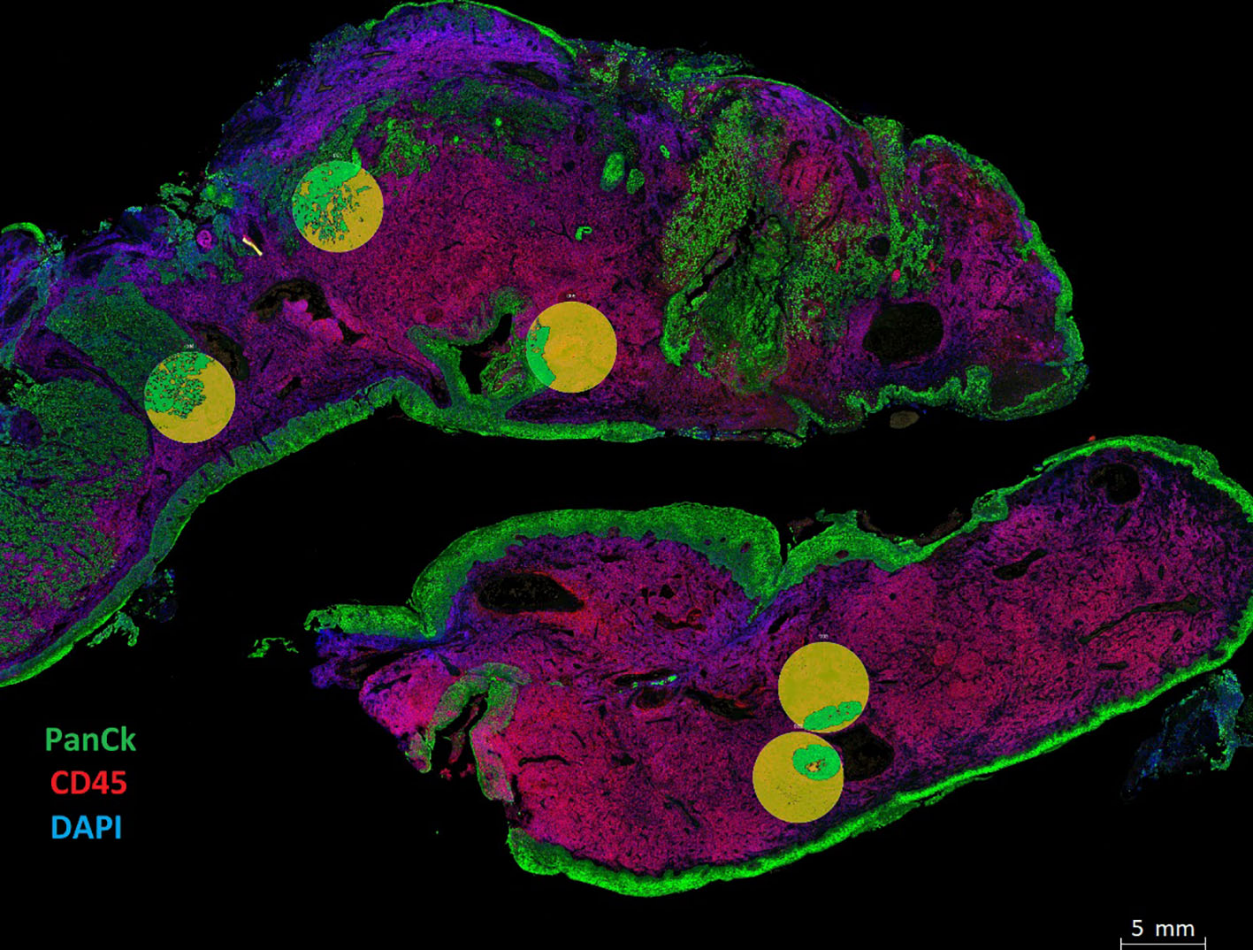
Spatial phenotyping on HNSCC tumor samples. Tissue compartmentalization was performed by masking panCK+ (Tumor) and panCK- (Stroma). The morphology markers included panCK (green) for tumor cells, CD45 (red) for immune cells, and SYTO 13 (blue) for the nucleus. Tumor segmentation on regions of interest (ROIs) was done to capture Tumor masks in green and Stromal regions in yellow. Masks were created using the panCK+/- feature to liberate barcodes for digital counting by the Nanostring nCounter platform.

The FFPE tissue slides were processed and analyzed by the Nanostring GeoMx Digital Spatial Profiler (DSP) technology at Queensland University of Technology (QUT), Brisbane, Australia. Fluorescent morphology markers, including CD45, pan-cytokeratin (panCK), and SYTO-13 were used to visualize lymphocytes, tumor regions, and the nucleus, respectively. The slides were prepared and processed, using an immune-oncology panel (**Table 2**), according to the manufacturer’s instructions. Tumor and stromal compartments were distinguished and defined by gating panCK regions and non-panCK regions, respectively (**Figure 2**). An immune-oncology panel of 68 oligonucleotide-conjugated primary antibodies, including the human immune cell core, immune cell typing, pan-tumor, immune activation status, immune-oncology (IO) drug target, cell death, and PI3K/AKT signaling panels, was employed to unravel protein expression of immune biomarkers (**Table 2**). Antibody barcodes were counted using the Nanostring nCounter platform according to the manufacturer’s instructions. External RNA Controls Consortium (ERCC) normalization and QC were applied in the DSP analysis suite, prior to data output for bioinformatics analysis.

**Table 2:** Nanostring Technologies GeoMx DSP Immuno-oncology panel.

Immune Cell Profiling	IO Drug Target / Immune Activation Status	Cell Death / PI3K-AKT Signalling	Immune Cell Typing / Pan-Tumour
Beta-2-microglobulin	4-1BB	BAD	CD14
CD11c	ARG1	BCL6	CD163
CD20	B7-H3	BCLXL	CD34
CD3	GITR	BIM	CD45RO
CD4	IDO1	CD95/Fas	CD66b
CD45	LAG3	GZMA	FAP-alpha
Ms IgG2a	OX40L	p53	FOXP3
Ms IgG1	STING	PARP	BCL-2
Rb IgG	TIM-3	Cleaved Caspase9	EpCAM
CD56	VISTA	Neurofibromin	ER alpha
GAPDH	CD127	Pan-AKT	HER2/ERBB2
SMA	CD25	MET	MART1
CD68	CD27	Phospho-AKT1 (S473)	NY-ESO-1
CD8	CD40	Phospho-GSK3B (S9)	PR
CTLA4	CD44	Phospho-Tuberin (T1462)	PTEN
Pan-Cytokeratin	CD80	Phospho-GSK3A (S21)/Phospho-GSK3B (S9)	S100B
Fibronectin	ICOS	INPP4B	
GZMB	PD-L2	PLCG1	
HLA-DR		Phospho-PRAS40 (T246)	
Ki-67			
Histone H3			
S6			
PD-1			
PD-L1			

### Data analysis (Nanostring GeoMx data)

Quality control (QC) of the data was performed using Bioconductor R package *standR* (v1.1.5). Filtering was first conducted to exclude slides and regions of interest (ROIs) with poor tissue quality due to poor staining or detachment. This resulted in having 17 patients tissue samples and 187 ROIs used for further analyses. Then, by using the function *addPerROIQC* from the *standR* package, marker LAG3 was filtered for being lowly expressed in over 90% of the ROIs. For data analysis, the log2-transformed count per million (logCPM) data were used to account for variation in library size. The relative log expression (RLE) and principal components analysis (PCA) of the logCPM data were used to assess the overall distribution, to identify any confounding factors from the experimental design and the detect presence of batch effects within the data. To remove unwanted technical variations observed in the RLE analysis, the data was normalized using size factor normalization methods in *standR* (v1.1.5).

Differential expression (DE) analysis was performed using R packages *edgeR* (v3.34.0) (11) and *limma* (3.48.0) (12). Briefly, DE was modelled using linear models with experimental factors as predictors. The variations in marker expressions were modelled as a combination of the common dispersion that applies to all genes and a marker-specific dispersion. To estimate the common and marker-specific variations, the variation of each marker was modelled by borrowing information from all other markers using an empirical Bayes approach (13). The linear model was then fitted to a given experimental design containing the biological factors of interest, patient variation and clinical information as covariates. DE was performed for specific contrasts of interest. The resulting statistic was an empirical Bayes moderated t-statistic. Multiple hypothesis testing adjustment was carried out with the Benjamini Hochberg procedure with an adjusted p-value < 0.05 used as the threshold to identify significant DE markers.

Survival analysis was performed by both Kaplan-Meier estimates using median protein expression for cohort stratification, and Cox proportional hazards model using continuous protein expression. Normalized replicate measurements in tumor/stroma compartment were averaged per patient for survival analysis. Survival analysis was performed using R package Survival (14) with the assistance of Queensland Cyber Infrastructure Foundation (QCIF, University of Queensland, Australia).

### PhenoCycler-Fusion (Akoya Biosciences)

Single-cell spatial phenotyping of the HNSCC FFPE slides was performed in collaboration with Akoya Biosciences (Massachusetts, US) on the PhenoCycler-Fusion platform. Four tissue slides from four different patients were stained with a 45-plex antibody panel, including immune checkpoints, immune cell lineage, activation states, general tissue structure in a single step (**Table 3**). Multiple combinations of three antibodies were visualized on the PhenoCycler-Fusion, by utilizing iterative fluorescent-reporter addition and image cycles. All experiments were performed according to manufacturer's instructions. Quality control of the data was performed qualitatively on each individual marker image and filtering was conducted to exclude markers and tissue regions with poor quality. Computational image analysis was used to identify cell types and characterize their spatial distribution in the tissue. The first step of image analysis consists of cell segmentation which is the step of identifying individual cells in the tissue, their surface, and their locations. Nuclear segmentation was first performed using StarDist (15) method applied to the DAPI channel, then cytoplasm segmentation was established by nuclear expansion using morphological dilation applied to the labelled nuclear mask, and the centroid of each cell was defined by the x-y coordinates in the image. The average intensity of each marker was then calculated for each cell from the corresponding expression compartment, e.g., nuclear surface, cytoplasmic surface, to produce an expression table where cells are listed with their protein expressions and locations in the tissue. Each protein expression was then z-scored across all the cells in the slide such that each protein has a mean equal to 0 and a standard deviation equal to 1. Then, the data from all slides were combined, resulting in a total of 3,106,317 cells. Batch correction was then performed using Harmony (16) based on 17 lineage markers (CD163, CD68, CD19, CD20, CD21, CD3e, CD8, CD4, CD45RO, ICOS, FOXP3, E-CAD, Pan-Cytokeratin, Beta-Catenin, CD31, CD34, SMA) in order to minimize batch effects between the tissue slides. The feature matrix returned by Harmony was then used for unsupervised clustering using Leiden algorithm (17) and the GPU-accelerated package, Rapids (18). Leiden resolution equals to 1-6 were tested and the resolution of 4 was chosen for manual cluster annotation as it presents a sufficient number of clusters that separate the cells into smaller groups (N = 70) differentiated by protein expression variability over the technical variability. Cell phenotypes were then assigned based on protein expression patterns on a hierarchical clustering heatmap. Clusters with similar expression profiles were combined into one phenotype and a new heatmap with 10 cell phenotypes was generated. Furthermore, tissue segmentation into 3 compartments was performed semi-automatically based on the Pan-Cytokeratin image. First, the tumor compartment was segmented by thresholding the image and applying morphological filters. Then, the tumor mask was dilated by 100um to delineate the “tumor front” compartment. The rest of the tissue was then labelled as stroma.

**Table 3:** Akoya Biosciences PhenoCycler-Fusion custom Immuno-oncology panel.

Immune Cell	Myeloid / Structural	Activity
CD4	CD163	PD1
CD68	CD11b	PDL1
CD20	MPO	ICOS
CD11c	iNOS	TIM3
CD8	panCK	LAG3
HLA-DR	E-cadherin	IDO1
Ki67	CD31	CD40
CD45RO	Podoplanin	HLA-E
CD3e	SMA	IFNG
CD44	Vimentin	VISTA
CD45	Collagen IV	CD15
HLA-A	CD34	CD21
CD14	B-catenin	Pax5
CD56		Granzyme B
CD19		CD38
		CD39
		TIGIT

## Results

### HNSCC cohort characteristics

This retrospective study spatially profiled HNSCC FFPE tissue samples from patients with metastatic HNSCC. The patients’ response to immunotherapy was categorized according to RECIST criteria. The study included patients with progressive disease (PD, n=8), stable disease (SD, n=3), partial response (PR, n=5), and complete response (CR, n=1). In terms of HPV status, n=7 patients were oropharyngeal p16 positive, n=3 patients were p16 negative, and n=7 patients were unknown (**Table 1**). FFPE tissues were pathology reviewed for tumor and stroma demarcation on H&E images.

### Identification of differentially expressed proteins using Nanostring GeoMx DSP

Using the Nanostring GeoMx DSP, we investigated the protein expression levels of 17 FFPE tissue samples from HNSCC patients. The tumor and stromal compartments were defined by masking high and low - panCK regions, measured against pathology H&E annotations, and the protein expression of each compartment was measured (**Figure 2**, circles defined by masking).

Expression matrix was evaluated by relative log expression (RLE) plots and PCA to identify confounding experimental effects. Technical variation was captured by RLE plot (**Supplementary Figure 1**), while in the PCA analysis, top 6 principal components explain 62.12% variation of the expression matrix (**Supplementary Figure 2**), and variations of both tumor stroma labelling and library size were observed on PC2 (**Supplementary Figure 2**). Normalization was then performed to remove unwanted technical variation such as library size with the assessment using RLE and PCA plots (**Supplementary Figures 1**, **2B**). The comparisons between Differential expression (DE) analysis were conducted on normalized protein counts to determine compartment-specific proteins associated with patient response groups.

### Differentially expressed proteins detected by the Digital Spatial Profiler (DSP)

**Figure 3:**
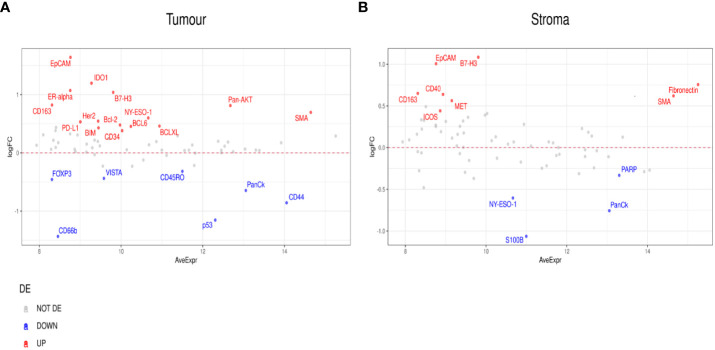
Differential protein expression between patient responders (n=9) and non-responders (n=8). **(A, B)** MA plots of Mean Expression (AveExpr) vs fold change (logFC) visualizing expression of protein biomarkers in responders compared to non-responders for either **(A)** tumor samples or **(B)** stroma samples. Colors denote not differentially expressed markers (gray), significantly up- (red) and down-regulated (blue) markers based on false discovery rate (FDR) < 0.05 after multiple comparison testing.

Differential expression was initially performed by grouping patients into those who responded to ICI treatment (CR, PR, SD) (n=9) and those who did not (PD) (n=8). This analysis indicated higher expression of PD-L1, B7-H3, Bcl-2, BCLX, and BIM in regions of tumors that responded to ICI. VISTA, CD45RO, CD66b, and FOXP3 were identified to have lower expression levels in regions of ICI responsive tumors (**Figure 3A**). In addition, the stromal compartment of patient-responders indicated increased expression of CD40, B7-H3, SMA, CD163, and ICOS, but decreased levels of PARP, NY-ESO-1, and S100B (**Figure 3B**).

**Figure 4:**
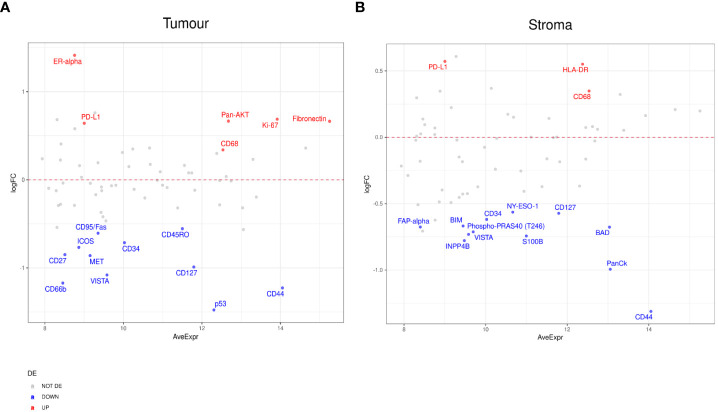
Differential protein expression between patients with partial response (PR) (n=5) and progressive disease (PD) (n=8). **(A, B)** MA plots of Mean Expression (AveExpr) vs fold change (logFC) visualizing expression of protein biomarkers in patients with PR compared to patients with PD for **(A)** tumor samples or **(B)** stromal samples. Colors denote not differentially expressed markers (gray), significantly up- (red) and down-regulated (blue) markers based on false discovery rate (FDR) < 0.05 after multiple comparison testing.

To further probe the factors associated with ICI response, we evaluated differential expression between RECIST subgroups, CR, PR, SD, PD. When we compared PR to PD, we found a number of significant differences. In the tumor compartment, patients with PR had higher expression of ER-alpha, PD-L1, Pan-AKT, CD68, Ki-67, and Fibronectin, but lower expression of CD44, CD127, CD34, and VISTA (**Figure 4A**). In the stromal compartment, patients with PR showed an increased expression of PD-L1, HLA-DR, and CD68, but decreased CD44, BIM, BAD, FAP-alpha, and VISTA expression levels when compared to patients with PD (**Figure 4B**).

**Figure 5:**
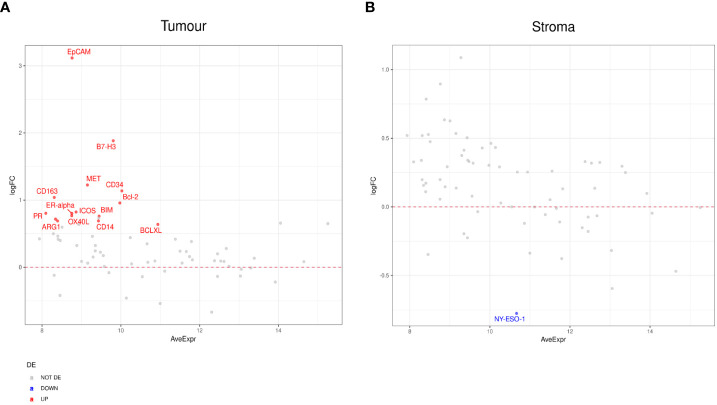
Differential protein expression between patients with stable disease (SD) (n=3) and progressive disease (PD) (n=8). **(A, B)** MA plots of Mean Expression (AveExpr) vs fold change (logFC) visualizing expression of protein biomarkers in patients with SD compared to patients with PD for **(A)** tumor samples or **(B)** stromal samples. Colors denote not differentially expressed markers (gray), significantly up- (red) and down-regulated (blue) markers based on false discover rate (FDR) < 0.05 after multiple comparison testing.

**Figure 6:**
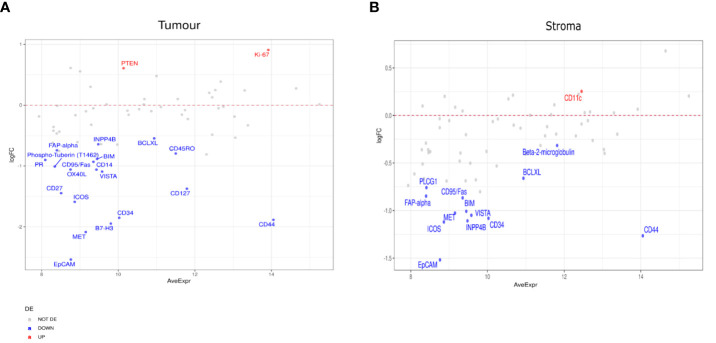
Differential protein expression between patients with partial response (PR) (n=5) and stable disease (SD) (n=3). **(A, B)** MA plots of Mean Expression (AveExpr) vs fold change (logFC) visualizing expression of protein biomarkers in patients with PR compared to patients with SD for **(A)** tumor samples or **(B)** stromal samples. Colors denote not differentially expressed markers (gray), significantly up- (red) and down-regulated (blue) markers based on false discovery rate (FDR) < 0.05 after multiple comparison testing.

In patients with SD compared to those with PD, we found markers including CD163, EpCAM, PR, and B7-H3, enriched in the tumor compartment of patients with SD (**Figure 5A**). In the stromal compartment, NY-ESO-1 was found to be the only DE protein between patients with SD and those with PD (**Figure 5B**). Differential expression analysis in the tumor compartment between patients with PR versus patients with SD, showed higher PTEN and Ki-67 expression in the former group, while the expression of immune markers, such as Fap-alpha, CD127, CD45RO, and CD27, were found to be decreased (**Figure 6A**). In the stroma, PR patients had higher levels of CD11c, but lower levels of proteins involved in cell death, such as BIM, CD95/Fas, and BCLXL (**Figure 6B**). The patient cohort included one patient with CR, which we compared to all the other response groups combined. Despite low statistical power due to a small sample size, a similar set of DE proteins were found in both the tumor and stroma of the patient with CR. The tumor region of the CR patient indicated higher levels of VISTA, CD45, IDO1, and S100B while S473 expression was found to be decreased. In the stroma, CD45, Bcl2, CD20, CD45RO, CD3, CD4, and HLA-DR were found to be higher, while the expression of Ki-67 was found to be lower (**Supplementary Figures 3A–F**).

**Figure 7:**
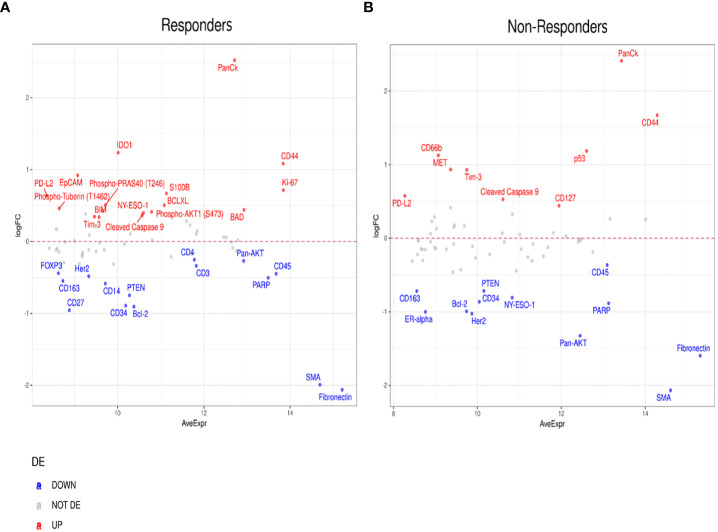
Differential protein expression between tumor and stromal compartments in different treatment outcomes. **(A, B)** MA plots of Mean Expression (AveExpr) vs fold change (logFC) visualizing expression of protein biomarkers in tumor compartment compared to stromal compartments in **(A)** responders or **(B)** non-responders. Colors denote not differentially expressed markers (gray), significantly up- (red) and down-regulated (blue) markers based on false discovery rate (FDR) < 0.05 after multiple comparison testing.

Furthermore, to investigate differentially expressed protein markers between the tumor and stromal margins, we performed a DE analysis in both patient responders and non-responders (**Figure 7**). We found that several protein biomarkers, most notably panCK, IDO1, CD44, and Ki-67, were expressed at higher levels in responders’ tumor compartments; however, in the stroma, SMA, Fibronectin, CD4, and CD27 were the most differentially expressed (**Figure 7A**). In the non-responder group, panCK, CD44 and CD66b were found to be the most differentially expressed proteins in the tumor compartment, while SMA and Fibronectin, and Pan-AKT were more highly expressed in the stroma (**Figure 7B**).

### Survival analysis

**Figure 8:**
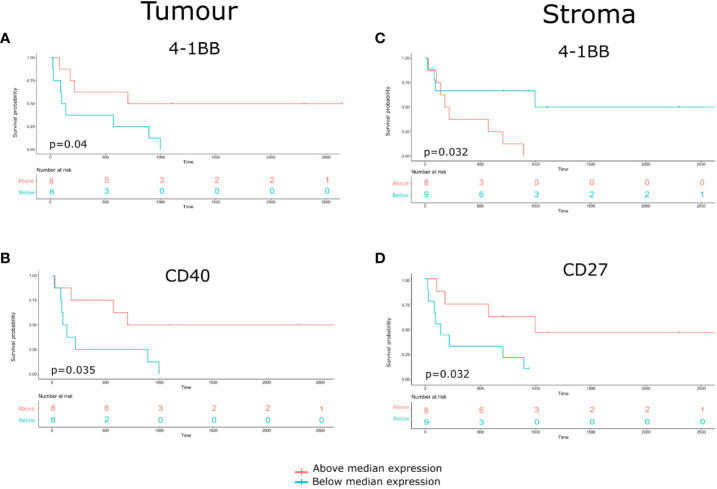
Identification of Compartmentalized protein enrichment with survival associations. **(A, B)** The Kaplan-Meier survival analyses conducted on the tumor protein biomarker expression. **(C, D)** The Kaplan-Meier survival analyses conducted on the stromal protein biomarker expression. The p-value of the curves is calculated using the log-rank test.

Compartmentalized protein expression was tested for associations with overall survival (OS). Protein expression was used to stratify the patient cohort into groups above and below the median threshold (**Figure 8** and **Supplementary Table 2**). This indicated that within tumor regions, high 4-1BB expression was associated with better survival (Log rank p= 0.04, Cox HR= 0.28) (**Figure 8A**), and high CD40 expression resulted in better survival (Log rank p= 0.035, Cox HR= 0.27) (**Figure 8B**). Additionally, within stromal regions, high 4-1BB expression was associated with poorer survival (Log rank p= 0.032, Cox HR= 2.24) (**Figure 8C**), while high CD27 expression was associated with better survival (Log rank p=0.032, Cox HR= 0.2) (**Figure 8D**).

Additionally, we performed a Kaplan-Meier survival analysis to look into the relationship between CD20 and overall survival. Despite a trend towards improved survival with higher CD20 expression, the result was not statistically significant due to the sample size limitation (**Supplementary Figure 4**). To investigate the association between patient subgroups and overall survival (OS), we conducted a COX proportional hazard model. We found that p16 positive status (a surrogate marker for HPV infection) was associated with a better survival (**Supplementary Figure 5**). Furthermore, a dendrogram heatmap to visualise the association between biomarker expression and patients’ clinical-pathological characteristics was constructed (**Supplementary Figure 6**).

### Single cell phenotyping of tissue samples with the Akoya PhenoCycler-Fusion

**Figure 9:**
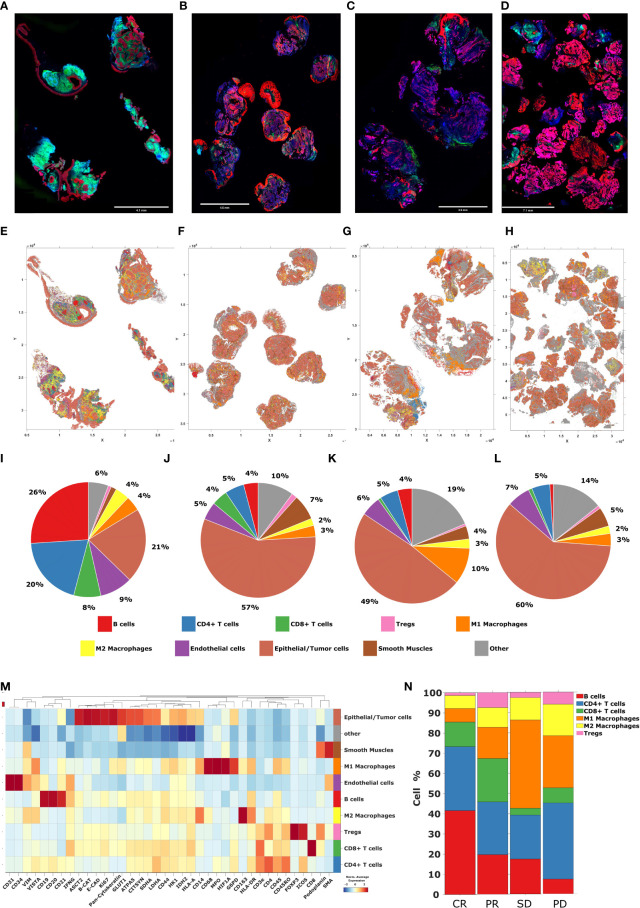
Cell phenotyping by Akoya PhenoCycler-Fusion. **(A–D)** High plex immunofluorescence (mIF) whole slide tissue images of patients with CR, PR, SD, and PD, from left to right respectively. Blue = DAPI, Green = CD45, Pink = E-cadherin, Red = panCK **(E–H)** Tissue maps of patients with CR, PR, SD, and PD, from left to right respectively. **(I–L)** The pie charts representing the abundance of distinct phenotype clusters sorted by color across tissue samples from patients with CR, PR, SD, and PD, from left to right respectively. **(M)** The heatmap including a curated clustering dendogram with cell types. **(N)** The bar chart representing cell type composition as well as the intra-tumor heterogeneity across tissue samples from patients with CR, PR, SD, and PD, from left to right respectively.

**Figure 10:**
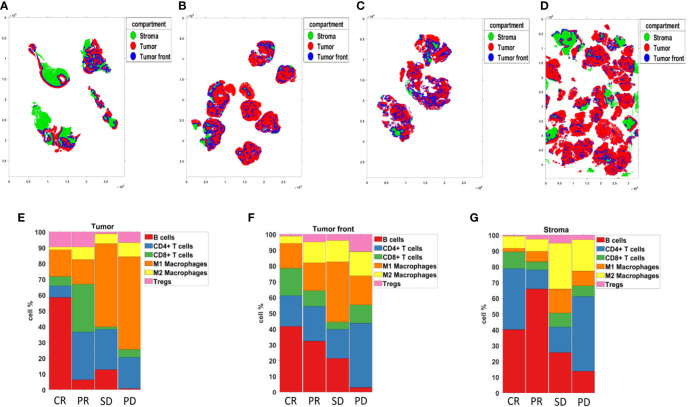
Compartmentalized analysis. **(A–D)** Tissue segmentation of patients with CR, PR, SD, and PD, from left to right respectively. **(E–G)** The bar chart representing Compartmentalized cell type composition across tissue samples from patients with CR, PR, SD, and PD, from left to right respectively.

Ultra-high plex immunofluorescence (IF) whole slide tissue images were obtained using the Akoya PhenoCycler-Fusion platform. The morphology markers included panCK (red), CD45 (green), DAPI (blue), and E-cadherin (pink) (**Figures 9A–D**). Cell type across different samples from patients with CR, PR, SD, and PD were generated (**Figures 9E–H**). Pie charts were constructed to reveal the abundance of distinct phenotype clusters across patients’ tissue samples (**Figures 9I–L**). A heatmap was created in order to show biomarker expression of different cell types (**Figure 9M**). To reveal the intra-tumor heterogeneity, a stacked bar graph indicating cell type composition was generated across patients’ tissue samples (**Figure 9N**). Additionally, tissue segmentation of the individual cases composed of clinical responses of CR, PR, SD, and PD was created to demonstrate the spatially resolved distribution of the tumor, tumor front, and stroma. (**Figures 10A–D**). Stacked bar graphs displaying cell type composition in various compartments was generated in these patients samples (**Figures 10E–G**). Most notably, the proportion of B-cells and CD8^+^ T-cells were highly enriched in the CR and PR groups compared to SD and PD, emphasizing a greater proportion of tumor B- and T-cell infiltration in the response groups.

## Discussion

Immune checkpoint inhibitors (ICIs), including anti-PD-1 antibodies Pembrolizumab and Nivolumab, have shown promising results in patients with R/M HNSCC However, only 15 to 20% of patients benefit from single-agent ICI (19). The immune characteristics of the TME play an important role in immunotherapy response, and include the cell types, densities, and their locations which influence the cell-cell communication involved in immune cell activity, and are therefore indicated as significant predictors of outcome to ICIs (20). Spatial analysis of the TME could help characterize the cellular and molecular interactions across tumor, immune, and non-immune cells. Watson et al., 2022 showed that the distance and proximity of immune cells, particularly CD8^+^ T cells, to tumor cells can influence immunotherapy response outcomes, supporting the significance of spatially resolved TME interrogation (10). In this study, we used two spatial profiling technologies to characterize HNSCC TME, the Nanostring GeoMx Digital Spatial Profiler (DSP) and the Akoya PhenoCycler-Fusion, to dissect the cellular architecture of ICI-treated patients to identify potential biomarkers or prognostic features from immunotherapy response groups. We first broadly grouped patients into Disease responders (CR, PR, SD) and non-responders (PD), and performed sub-analysis of these individual groups. We found that immune checkpoint molecules and tumor necrosis factor receptor (TNFR) superfamily members were indicative of response to immunotherapy.

It is well established that after T cell activation, T cells express inhibitory cell surface receptors, known as immune checkpoint proteins, such as program cell death-1 (PD-1), to prevent further activation. However, tumor cells exploit this process by expressing ligands for these receptors, for example programmed death-ligand 1 (PD-L1 or B7-H1) to bind PD-1, in order to cause T cell exhaustion, which results in T cell inactivation and reduced effector function (21). Studies have shown that PD-L1 expression on tumors or immune cells such as macrophages could be a predictive biomarker of immunotherapy response (6, 22). Similarly, we found PD-L1 higher expression in the tumor regions of responders relative to non-responders. In addition, we found that patients with PR had higher levels of PD-1 and PD-L1 expression in tumor regions than those with PD, supporting the role and benefit of PD-1/PD-L1 axis blockade in HNSCC. Another member of B7 ligand family B7-H3, also known as CD276, is an immune checkpoint inhibitor molecule that regulates the immune system (23). B7-H3 is found to impair natural killer (NK) cell, CD4 T, and CD8 T cell effector functions by releasing a variety of cytokines (23). B7-H3 expression may be linked to immune suppression, which promotes tumor cell growth. Recent studies have found that in a number of solid tumors, including breast, ovarian, brain, and gastric cancer, as well as Merkel cell carcinoma, showed that high expression of B7-H3 increased tumor size, invasion, and proliferation, which were linked to worse prognosis and overall survival (24). However, we found higher levels of B7-H3 expression in both the tumor and stromal compartments of responders compared to non-responders. This finding is consistent with our previous published work on oropharyngeal squamous cell carcinoma (OPSCC), in which we demonstrated that B7-H3 expression in the stroma of nodal metastatic samples was associated with improved overall survival (OS) in patients (25). Here, we show that V-domain immunoglobulin suppressor of T cell activation (VISTA) was enriched in non-responders. VISTA is an inhibitory checkpoint protein belongs to the B7 family that impedes T cell activation and proliferation (26). By being expressed as a ligand on the antigen presenting cells (APCs) or as an inhibitory receptor on T cells, VISTA could play the inhibitory roles (27). Recent studies show that VISTA expression on tumor cells, such as ovarian and endometrial cells, suppress T cell cytokine production and also infiltration (28). In animal immunotherapy studies, therapies targeting both VISTA and PD-L1 proteins could lead to tumor regression and improved survival (29, 30). Furthermore, VISTA was reported to be highly expressed in FOXP3 Tregs and associated with the suppression of the immune system (31, 32). Accordingly, in our study, the expression of VISTA, as well as FOXP3, was lower in tumor regions of responders relative to non-responders.

TNFR superfamily consists of 30 receptors and 19 ligands, all of which have been shown to be involved in cell signaling pathways, influencing the differentiation, proliferation, and survival of immune and non-immune cells (33, 34). In this study, we discovered several TNFR superfamily members, including CD40, OX40L, CD27, 4-1BB, and CD95/Fas, which were differentially expressed by response groups and also associated with overall survival (OS) (35). CD40 (TNFRSF5) is a co-stimulatory cell surface receptor expressed in both hematopoietic and non-hematopoietic cells, where it can activate DCs, prime tumor-specific CD8 T cells, and finally stimulate antitumor immunity (36). CD40 was also shown to promote tumor regression by directing macrophages to infiltrate tumors and increasing the expression of matrix metalloproteinases (37). Accordingly, here we found that CD40 had higher expression in the stroma of responders relative to non-responders. Furthermore, survival analysis revealed that higher CD40 expression was associated with better survival.

OX40L (also known as CD252) is a type II glycoprotein that is expressed in a variety of cell types, including B cells, macrophages, and dendritic cells (DCs), and is involved in multiple T cell subtype activation (38). Studies have shown that targeting OX40-OX40L interactions may increase CD4 and CD8 T cell survival, resulting in a better response to immunotherapy and a better patient prognosis (39, 40). Here, we showed that patients with SD had higher levels of tumor OX40L expression than those with progression. Cluster of differentiation 27 (CD27) is a costimulatory T cell receptor that plays a role in T cell proliferation and differentiation into memory and effector T cells (41). CD27 is known to have immune suppressive effects by increasing Treg survival and inducing effector T cell apoptosis (42, 43). Similarly, we found that patients with PR had lower levels of CD27 in the tumor compartment relative to those with PD. However, CD27 expression in the stroma was associated with better survival. In addition, when we compared protein biomarker expression between tumor and stromal compartments, we found that CD27 had a higher expression level in the stromal margins of patient responders. Taking together, we found that the expression of CD27 in the stroma, but not in the tumor, might be associated with a better prognosis in patients with HNSCC.

4-1BB (CD137), a surface glycoprotein and costimulatory receptor expressed on activated T cells, was found to boost cytokine secretion, antiapoptotic molecule upregulation, and T cell effector function (44). Published work indicates that therapies containing 4-1BB result in tumor regression (45). In our study, we showed that higher expression of 4-1BB in the tumor compartment, but not in the stroma, was associated with better survival in HNSCC patients. CD95/Fas is a death receptor that binds to its ligand CD95L/FasL to initiate a death signaling pathway (46). Aside from apoptotic functions, CD95/Fas has been shown to activate anti-apoptotic signaling pathways such as JNK, MAPKs and NF-kβ, resulting in cell survival, proliferation, and migration (47). Qadir et al, have reported that tumor cells express CD95/Fas in order to support their growth (48). Here, we found that patients with PR had lower expression levels of CD95/Fas in the tumor compartment compared to patients with progression. However, in a comparison of CD95/Fas protein expression in the patient with CR versus those with PR, this protein was found to be more abundant in the stromal compartment of CR, despite low statistical power.

Co-stimulatory molecules, such as CD40 or 4-1BB, have positive impact on immune cell functions, but co-inhibitory molecules, such as PD-1/PD-L1 or CTLA-4, have the opposite effect (49, 50). Therefore, targeting these signaling molecules together, which means agonistic antibodies for TNFR molecules along with antagonistic antibodies for ICIs, could improve immune activation and anti-tumor immune responses (49, 51). It was found that the combination of CTLA-4 blockade and 4-1BB co-stimulation could increase tumor infiltrating lymphocytes, such as CD4^+^ and CD8^+^ T cells, contributing to further tumor eradication and, eventually, survival improvement (51–53). Also, the combination of agonistic anti-4-1BB antibodies and anti-PD-1 plus radiotherapy demonstrated promising antitumor activity (51, 54, 55).

Due to the limitations of ROI-based data generation, which collects protein expression only in ROI selected areas rather than resolved singe-cell resolution, we employed the Akoya PhenoCycler-Fusion on a representative whole section tissue sample for each response group. The PhenoCycler-Fusion data identified an increased presence of B-cells and CD8^+^ T-cells in patients with CR/PR compared to SD/PD (**Figures 9**, **10**). A similar trend of an increased CD20 (B-lymphocyte antigen) was found using the Nanostring GeoMx ROI based approach in the CR group. The presence of B-cells, and mature tertiary lymphoid structures in the tumor microenvironment has been reported to be favorable for immunotherapy treatment (56, 57). In this approach, high-plex single-cell based approaches have been used to validate the ROI selection strategy.

## Conclusion

In this study of patients with R/M HNSCC, we found that Compartmentalized expression of immune checkpoint molecules may stratify patient responders from non-responders to immunotherapy. Moreover, when we further performed sub-analysis of the clinical groupings, we found that TNFR superfamily of molecules were important factors of response to immunotherapy. These proteins drive a wide range of functions, including dichotomous roles of T cell activation and cell death signaling. Taken together, our study demonstrates how targeted spatial proteomic approaches may provide new cues to identify biomarkers of ICI therapy in HNSCC. Moreover, these data may aid in the identification of new Compartmentalized biomarkers for routine clinical use by standard pathology immunohistochemistry staining.

## Data availability statement

The datasets presented in this study can be found in online repositories. The names of the repository/repositories and accession number(s) can be found below: GSE217640 (GEO).

## Ethics statement

This study has Human Research Ethics (HREC) approval from the Royal Brisbane and Women’s Hospital (RBWH) (LNR/2020/QRBW/66744) and The University of Queensland. The patients/participants provided their written informed consent to participate in this study.

## Author contributions

Idea concept, BH, KO’B, and AK. Methodology/experimental, HS, JM, NM, MW, MA, QN, RL, OB, BH, and AK. Data analysis, NL, JM, BC, NJ, CT, OB, and MD. Writing and critical review, all authors. All authors contributed to the article and approved the submitted version.

## Supplementary material

Supplementary Figure 1 Data normalization by relative log expression (RLE). **(A, B)** Relative log expression (RLE) per regions of interest (ROIs) were constructed before and after normalization to evaluate confounding experimental effects.

Supplementary Figure 2 Dimensional reduction analysis using principal component analysis (PCA). **(A, B)** Principal component analysis (PCA) plots were constructed to visualize batch effects before and after normalization. **(C)** Scree plot visualizing the number of the principal component against its corresponding eigenvalue.

Supplementary Figure 3 Differential protein expression between patients with complete response (CR) compared to all other response groups. **(A)** Limma-voom MA plot demonstrating tumor expression of protein biomarkers in patients with CR compared to patients with PR, ranked by fold change (logFC). **(B)** Limma-voom MA plot demonstrating stromal expression of protein biomarkers in patients with CR compared to patients with PR, ranked by fold change (logFC). **(C)** Limma-voom MA plot demonstrating tumor expression of protein biomarkers in patients with CR compared to patients with SD, ranked by fold change (logFC). **(D)** Limma-voom MA plot demonstrating stromal expression of protein biomarkers in patients with CR compared to patients with SD, ranked by fold change (logFC). **(E)** Limma-voom MA plot demonstrating tumor expression of protein biomarkers in patients with CR compared to patients with PD, ranked by fold change (logFC). **(F)** Limma-voom MA plot demonstrating stromal expression of protein biomarkers in patients with CR compared to patients with PD, ranked by fold change (logFC).

Supplementary Figure 4 CD20 expression and association with survival. Kaplan-Meier survival curve visualizing the relationship between CD20 expression of patients’ survival.

Supplementary Figure 5 Identification of patients’ subgroup with overall survival associations. Multivariate COX proportional hazard analysis is conducted to show the hazard ratio with 95% confidence interval for different subgroup of patients. HR>1 indicates association with poorer outcomes.

Supplementary Figure 6 Dendrogram heatmap of patients grouped by best responses in columns and protein expression in rows. **(A)** Dendrogram of tumor protein expression. **(B)** Dendrogram of stromal protein expression. Complete Response (green), Partial response (orange), stable disease (purple), progressive disease (blue).
