# Supplementary figures and images for: Compartmentalized spatial profiling of the tumor microenvironment in head and neck squamous cell carcinoma identifies immune checkpoint molecules and tumor necrosis factor receptor superfamily members as biomarkers of response to immunotherapy
Source: Front Immunol. 2023 Apr 3;14:1135489. doi: 10.3389/fimmu.2023.1135489 (PMC10154785; doi:10.3389/fimmu.2023.1135489)

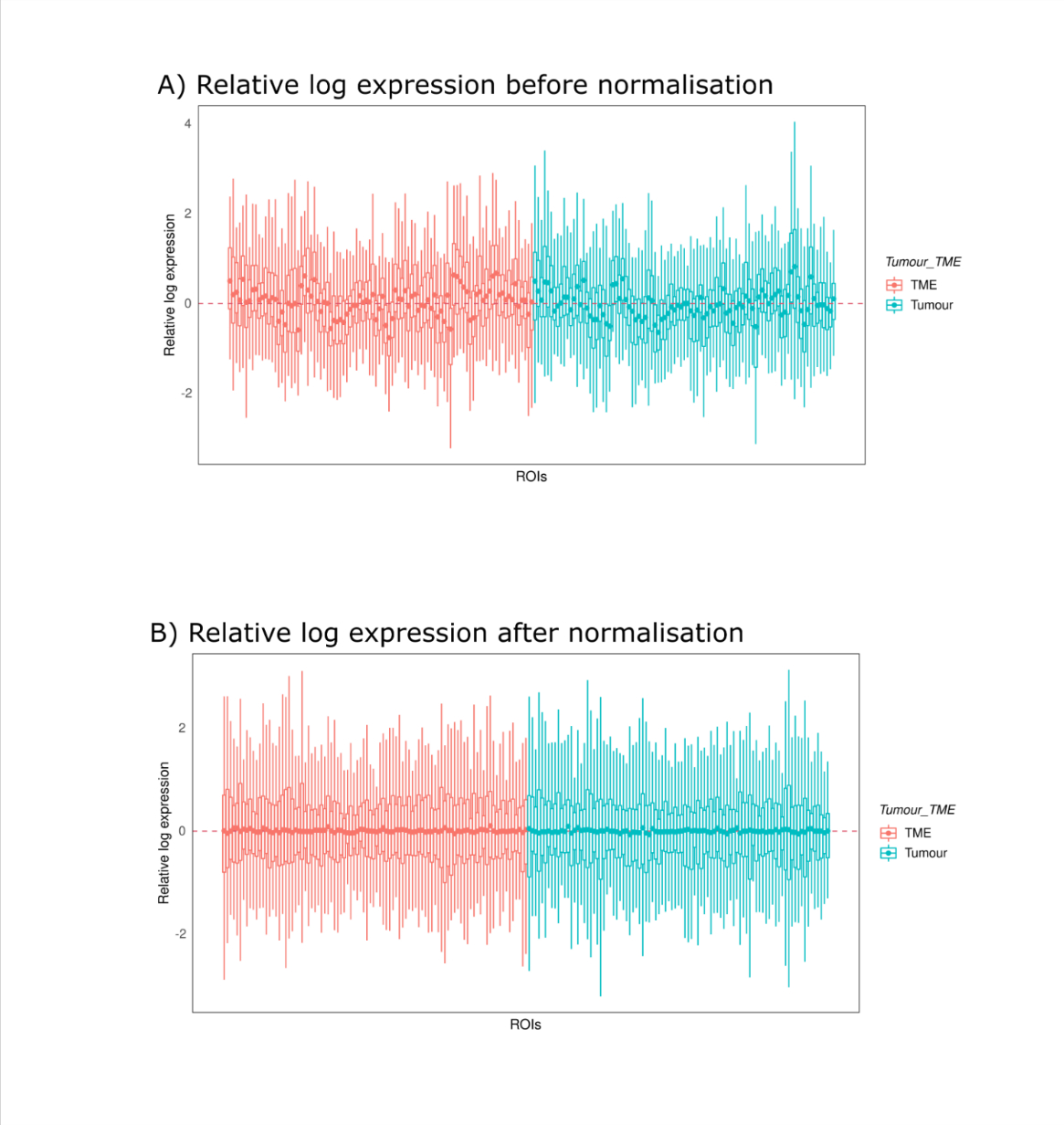

Supplement: Supplementary Figure 1 — Data normalization by relative log expression (RLE). (A, B) Relative log expression (RLE) per regions of interest (ROIs) were constructed before and after normalization to evaluate confounding experimental effects. [file Image_1.jpg]

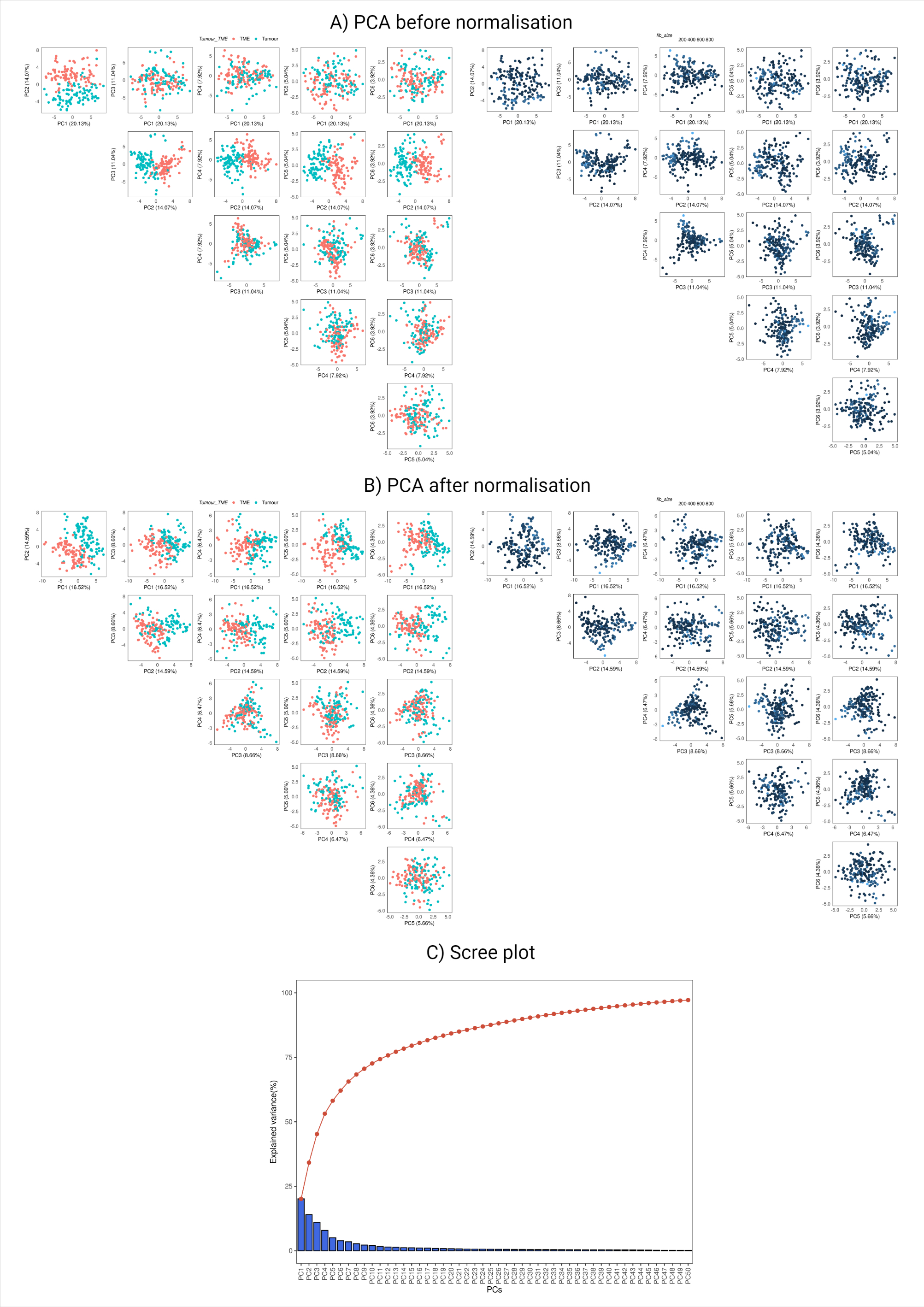

Supplement: Supplementary Figure 2 — Dimensional reduction analysis using principal component analysis (PCA). (A, B) Principal component analysis (PCA) plots were constructed to visualize batch effects before and after normalization. (C) Scree plot visualizing the number of the principal component against its corresponding eigenvalue. [file Image_2.jpg]

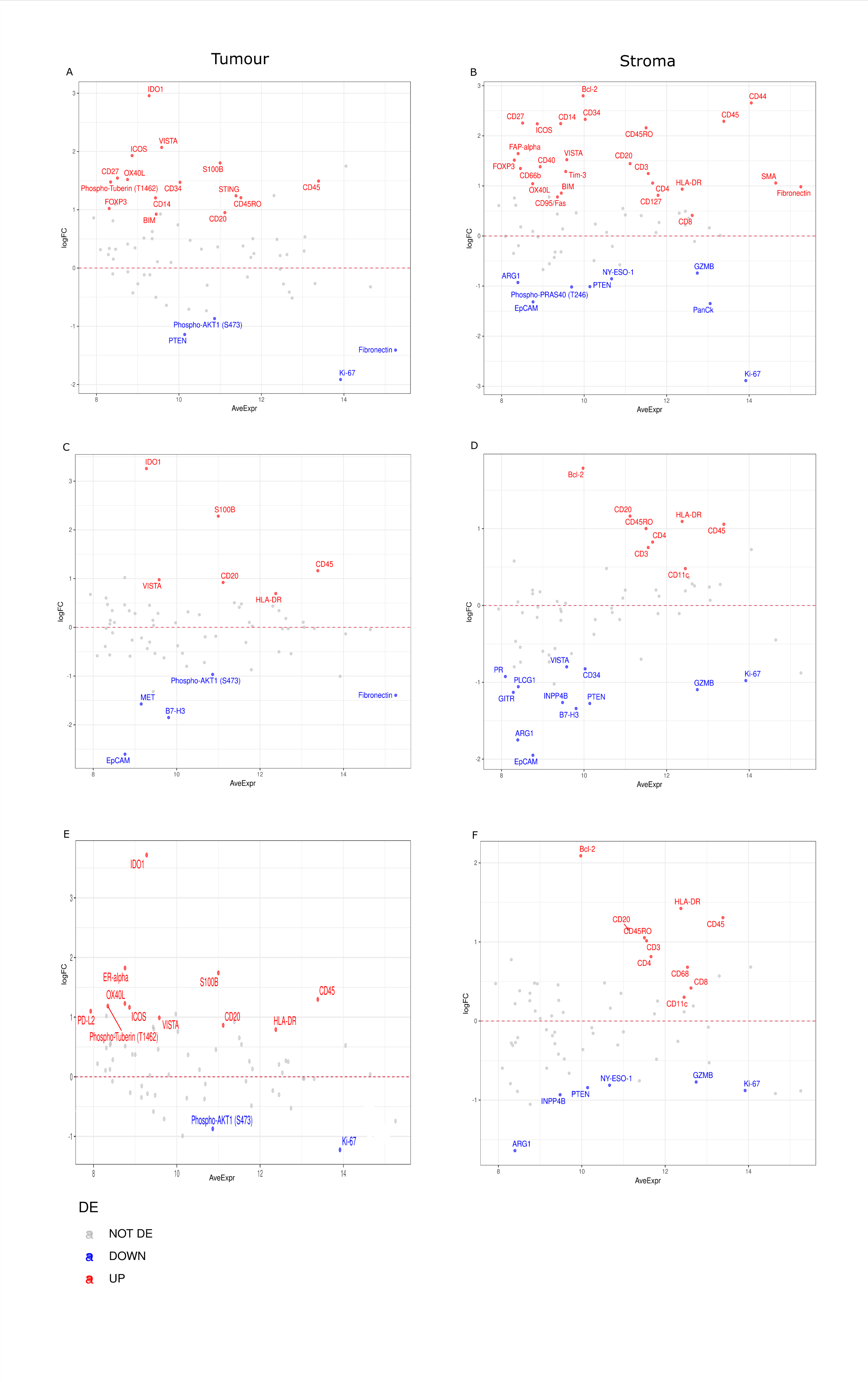

Supplement: Supplementary Figure 3 — Differential protein expression between patients with complete response (CR) compared to all other response groups. (A) Limma-voom MA plot demonstrating tumor expression of protein biomarkers in patients with CR compared to patients with PR, ranked by fold change (logFC). (B) Limma-voom MA plot demonstrating stromal expression of protein biomarkers in patients with CR compared to patients with PR, ranked by fold change (logFC). (C) Limma-voom MA plot demonstrating tumor expression of protein biomarkers in patients with CR compared to patients with SD, ranked by fold change (logFC). (D) Limma-voom MA plot demonstrating stromal expression of protein biomarkers in patients with CR compared to patients with SD, ranked by fold change (logFC). (E) Limma-voom MA plot demonstrating tumor expression of protein biomarkers in patients with CR compared to patients with PD, ranked by fold change (logFC). (F) Limma-voom MA plot demonstrating stromal expression of protein biomarkers in patients with CR compared to patients with PD, ranked by fold change (logFC). [file Image_3.jpg]

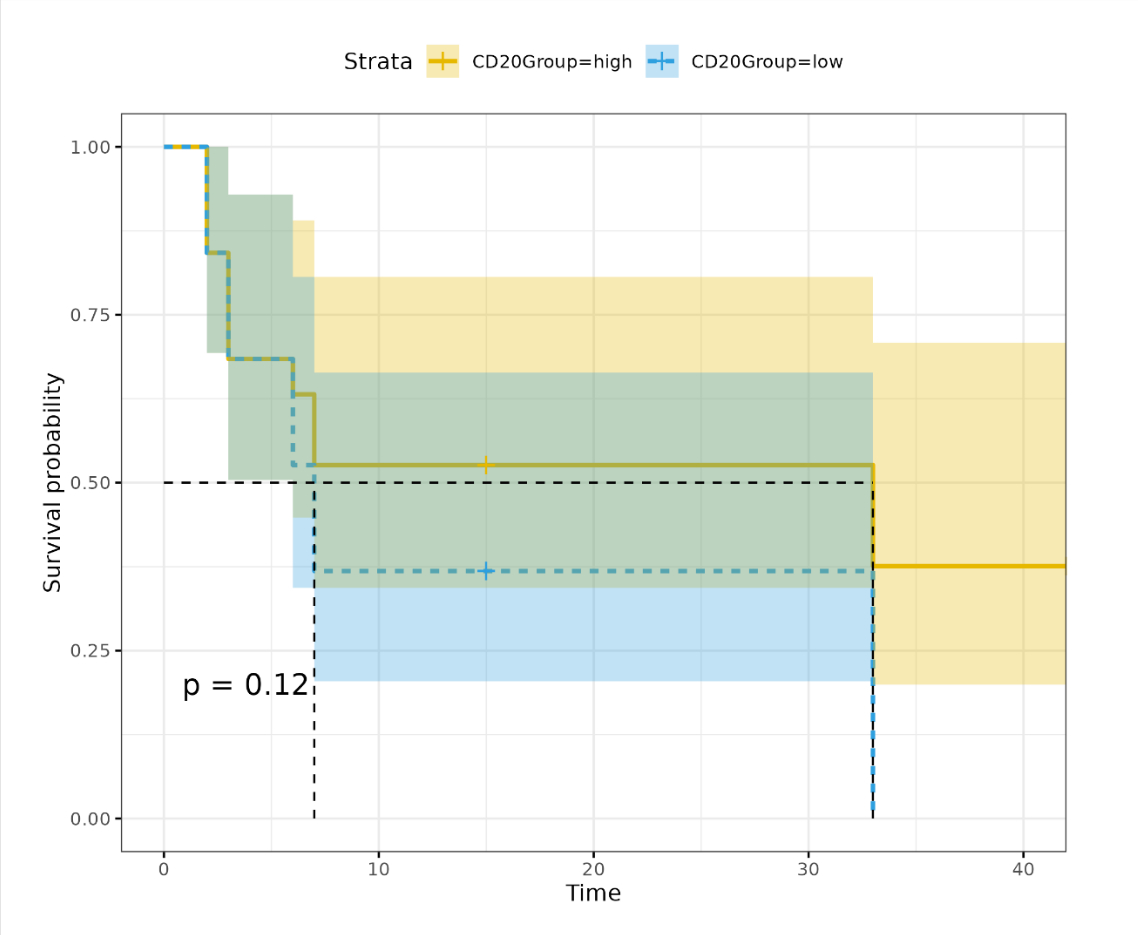

Supplement: Supplementary Figure 4 — CD20 expression and association with survival. Kaplan-Meier survival curve visualizing the relationship between CD20 expression of patients’ survival. [file Image_4.jpg]

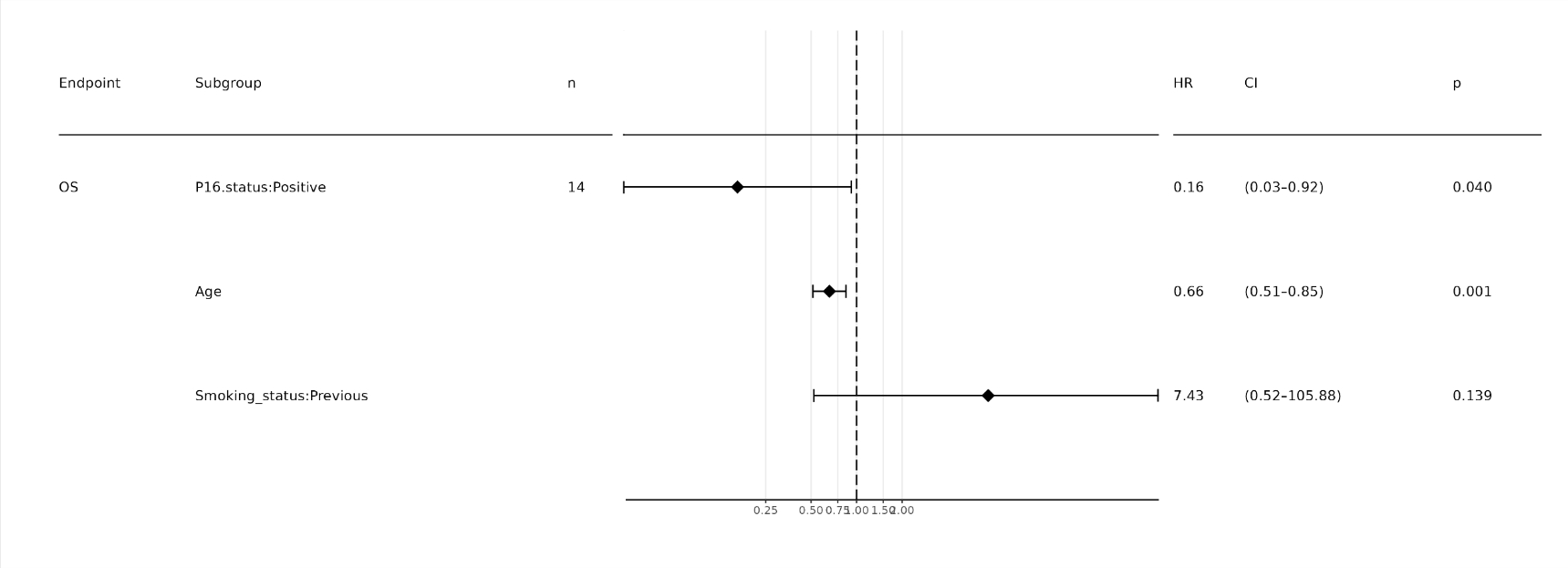

Supplement: Supplementary Figure 5 — Identification of patients’ subgroup with overall survival associations. Multivariate COX proportional hazard analysis is conducted to show the hazard ratio with 95% confidence interval for different subgroup of patients. HR>1 indicates association with poorer outcomes. [file Image_5.jpg]

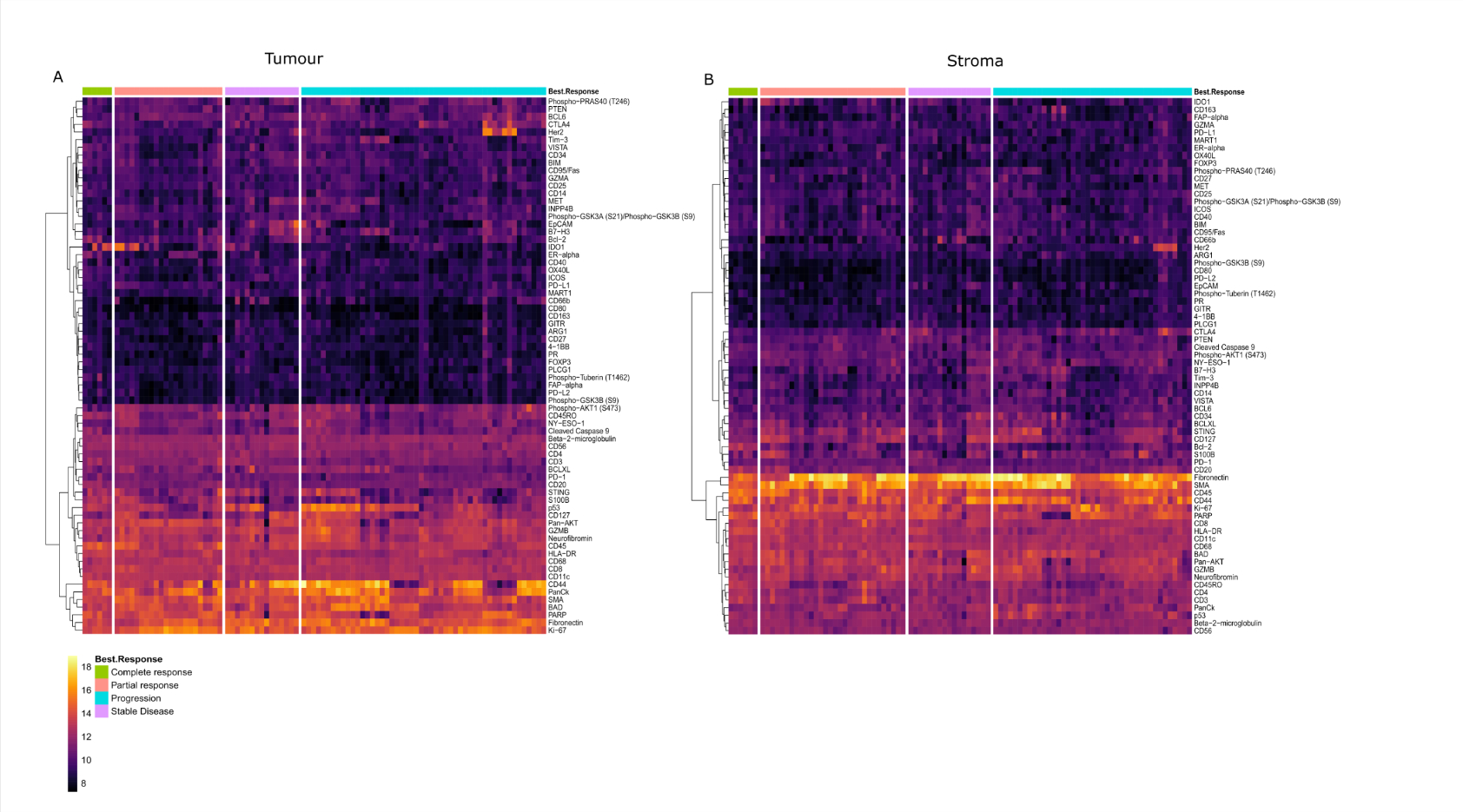

Supplement: Supplementary Figure 6 — Dendrogram heatmap of patients grouped by best responses in columns and protein expression in rows. (A) Dendrogram of tumor protein expression. (B) Dendrogram of stromal protein expression. Complete Response (green), Partial response (orange), stable disease (purple), progressive disease (blue). [file Image_6.jpg]
